# Retraction: Akt inhibition enhanced the growth inhibition effect of low-dose heavy-ion radiation via the PI3K/Akt/P53 signaling pathway in C6 glioblastoma cells

**DOI:** 10.3389/fonc.2023.1205993

**Published:** 2023-05-25

**Authors:** Frontiers Editorial Office

The journal retracts the April 1, 2021 article cited above.

Following publication, concerns were raised regarding the integrity of the images in the published figures. The authors failed to provide the raw data or a satisfactory explanation during the investigation, which was conducted in accordance with Frontiers’ policies. Given the concerns about the validity of the data, and the lack of raw data, the editors no longer have confidence in the findings presented in the article.

This retraction was approved by the Chief Editors of Frontiers in Oncology.

The authors did not agree to this retraction.

